# Association of candidate genetic variants and circulating levels of ApoE/ApoJ with common neuroimaging features of cerebral amyloid angiopathy

**DOI:** 10.3389/fnagi.2023.1134399

**Published:** 2023-04-11

**Authors:** Anna Bonaterra-Pastra, Sònia Benítez, Olalla Pancorbo, David Rodríguez-Luna, Carla Vert, Alex Rovira, M. Mar Freijo, Silvia Tur, Maite Martínez-Zabaleta, Pere Cardona Portela, Rocío Vera, Lucia Lebrato-Hernández, Juan F. Arenillas, Soledad Pérez-Sánchez, Ana Domínguez-Mayoral, Joan Martí Fàbregas, Gerard Mauri, Joan Montaner, Jose Luis Sánchez-Quesada, Mar Hernández-Guillamon

**Affiliations:** ^1^Neurovascular Research Laboratory, Vall d’Hebron Research Institute, Universitat Autònoma de Barcelona, Barcelona, Spain; ^2^Cardiovascular Biochemistry Group, Research Institute of the Hospital de Sant Pau (IIB Sant Pau), Barcelona, Spain; ^3^Center for Biomedical Research Network on Diabetes and Associated Metabolic Diseases (CIBERDEM), Instituto de Salud Carlos III (ISCIII), Madrid, Spain; ^4^Stroke Research Group, Vall d’Hebron Research Institute, Barcelona, Spain; ^5^Section of Neuroradiology, Department of Radiology, Vall d’Hebron University Hospital, Vall d’Hebron Research Institute, Universitat Autònoma de Barcelona, Barcelona, Spain; ^6^Neurovascular Group, BioCruces Health Research Institute, Barakaldo, Spain; ^7^Department of Neurology, Son Espases University Hospital, Balearic Islands, Spain; ^8^Department of Neurology, Donostia University Hospital, San Sebastián, Spain; ^9^Department of Neurology, Bellvitge University Hospital, L’Hospitalet de Llobregat, Spain; ^10^Stroke Unit, Department of Neurology, Ramón y Cajal University Hospital, Madrid, Spain; ^11^Stroke Unit, Department of Neurology and Neurophysiology, Virgen del Rocío University Hospital, Seville, Spain; ^12^Stroke Program, Department of Neurology, Hospital Clínico Universitario, Valladolid, Spain; ^13^Clinical Neurosciences Research Group, Department of Medicine, University of Valladolid, Valladolid, Spain; ^14^Department of Neurology, Virgen Macarena University Hospital, Seville, Spain; ^15^Stroke Unit, Department of Neurology, Hospital de la Santa Creu i Sant Pau, Barcelona, Spain; ^16^Stroke Unit, Department of Neurology, Hospital Universitari Arnau de Vilanova de Lleida, Lleida, Spain; ^17^Stroke Research Program, Institute of Biomedicine of Seville (IBiS), Virgen del Rocío University Hospital, University of Seville, Seville, Spain; ^18^Department of Neurology, Virgen Macarena University Hospital, Seville, Spain

**Keywords:** ApoE, ApoJ, CAA, MRI, lipoproteins, EPVS

## Abstract

**Introduction:**

Cerebral amyloid angiopathy (CAA) is characterized by the accumulation of amyloid-β (Aβ) in brain vessels and is a main cause of lobar intracerebral hemorrhage (ICH) in the elderly. CAA is associated with magnetic resonance imaging (MRI) markers of small vessel disease (SVD). Since Aβ is also accumulated in Alzheimer’s disease (AD) in the brain parenchyma, we aimed to study if several single nucleotide polymorphisms (SNPs) previously associated with AD were also associated with CAA pathology. Furthermore, we also studied the influence of APOE and CLU genetic variants in apolipoprotein E (ApoE) and clusterin/apolipoprotein J (ApoJ) circulating levels and their distribution among lipoproteins.

**Methods:**

The study was carried out in a multicentric cohort of 126 patients with lobar ICH and clinical suspicion of CAA.

**Results:**

We observed several SNPs associated with CAA neuroimaging MRI markers [cortical superficial siderosis (cSS), enlarged perivascular spaces in the centrum semiovale (CSO-EPVS), lobar cerebral microbleeds (CMB), white matter hyperintensities (WMH), corticosubcortical atrophy and CAA-SVD burden score]. Concretely, ABCA7 (rs3764650), CLU (rs9331896 and rs933188), EPHA1 (rs11767557), and TREML2 (rs3747742) were significantly associated with a CAA-SVD burden score. Regarding circulating levels of apolipoproteins, protective AD SNPs of CLU [rs11136000 (T) and rs9331896 (C)] were significantly associated with higher HDL ApoJ content in the lobar ICH cohort. APOEε2 carriers presented higher plasma and LDL-associated ApoE levels whereas APOEε4 carriers presented lower plasma ApoE levels. Additionally, we observed that lower circulating ApoJ and ApoE levels were significantly associated with CAA-related MRI markers. More specifically, lower LDL-associated ApoJ and plasma and HDL-associated ApoE levels were significantly associated with CSO-EPVS, lower ApoJ content in HDL with brain atrophy and lower ApoE content in LDL with the extent of cSS.

**Discussion:**

This study reinforces the relevance of lipid metabolism in CAA and cerebrovascular functionality. We propose that ApoJ and ApoE distribution among lipoproteins may be associated with pathological features related to CAA with higher ApoE and ApoJ levels in HDL possibly enhancing atheroprotective, antioxidative, and anti-inflammatory responses in cerebral β-amyloidosis.

## 1. Introduction

Cerebral amyloid angiopathy (CAA) is a degenerative small vessel disease (SVD) caused by the deposition of amyloid, commonly amyloid-β (Aβ), in the wall of the blood vessels of the central nervous system, affecting leptomeningeal vessels, arteries and cortical arterioles of medium or small caliber, as well as cerebral capillaries (Charidimou et al., 2017a). The most relevant pathological consequence of CAA is the presence of intracerebral hemorrhage (ICH) in cortical and subcortical localization with ICH recurrence being a common complication of this disease (Charidimou et al., 2017d). Cognitive deterioration, dementia and transient focal neurologic episodes (TFNE) are also relevant clinical manifestations of CAA (Arvanitakis et al., 2011; Smith et al., 2021). Symptomatic CAA is characteristically associated with magnetic resonance imaging (MRI) markers of small vessel brain injury, including hemorrhagic markers as lobar cerebral microbleeds (CMB) and cortical superficial siderosis (cSS) (Charidimou et al., 2017c,d), but also non-hemorrhagic features of SVD, such as enlarged perivascular spaces in the centrum semiovale (CSO-EPVS) (Charidimou et al., 2017b) and periventricular white matter hyperintensities (WMH) (Chojdak-Łukasiewicz et al., 2021). These imaging features together with clinical characteristics are currently used to diagnose patients as possible or probable CAA according to the modified Boston criteria (Linn et al., 2010), which have recently been validated and updated (Charidimou et al., 2022). In parallel, a SVD score obtained from some of these common CAA MRI features, previously validated and associated with pathological CAA, has been defined to predict the vascular affectation degree in this disease (Charidimou et al., 2016; Boulouis et al., 2017).

Pathologically, CAA frequently coexists with Alzheimer’s disease (AD), where Aβ is accumulated in the brain parenchyma forming insoluble plaques. Therefore, both CAA and AD are grouped as cerebral β-amyloidosis diseases. In addition, AD brains present neurofibrillary tangles (NFT) formed by insoluble hyperphosphorylated Tau, that together with the presence of Aβ plaques induce neurodegenerative processes causing dementia (Scheltens et al., 2021). Besides the biological overlap between AD and CAA, based on Aβ production and clearance processes, different factors are described to favor the Aβ accumulation in different brain localizations (Greenberg et al., 2020). For example, the length of the peptide is clearly related to its deposition: Aβ42 is the main component of amyloid plaques in AD, whereas Aβ40 is predominantly aggregated in blood vessels (Yamada, 2015). Apolipoprotein E (ApoE), which has the main function of regulating lipids transport and distribution in the brain (Bu, 2010), is also a crucial element associated with Aβ brain deposition. This last assumption is based on the fact that the ε4 variant of the APOE gene is the main genetic risk factor for AD (Schmechel et al., 1993), but also for CAA (Greenberg et al., 1996). Indeed, APOEε4 is related not only to CAA but also to lobar ICH and lobar microhemorrhages, characteristic traits in CAA patients (Inoue et al., 2021). On the other hand, the APOEε2 variant is more frequent in brains with more severe vascular changes and it is associated with the presence and recurrence of ICH attributed to CAA (Greenberg et al., 1998). However, while both APOEε2 and APOEε4 alleles are considered risk factors for ICH-CAA (Giralt-Steinhauer et al., 2022), APOEε2 is a protective factor for AD (Serrano-Pozo et al., 2021). On the other hand, apolipoprotein J (ApoJ) or clusterin (CLU) coded by the CLU gene, is the second most expressed apolipoprotein in the brain after ApoE. Both ApoE and ApoJ are important factors in lipid metabolism that can also modulate Aβ aggregation and deposition in the brain (Huynh et al., 2017; Foster et al., 2019). The distribution of these apolipoproteins among different plasma lipoproteins can play a role in their functionality (Koch and Jensen, 2016; Lanfranco et al., 2020). In this regard, we have previously described how the content of ApoE and ApoJ in lipoproteins differs between CAA-ICH and AD patients (Bonaterra-Pastra et al., 2021).

To further understand the rationale underlying cerebral β-amyloidosis and the biological relationship between CAA and AD, our study was based on the determination of the frequency of different genetic variants previously associated with AD pathology in a cohort of patients with lobar ICH associated with CAA. In this sense, candidate single nucleotide polymorphisms (SNPs) were selected for being previously associated with AD occurrence and progression in different GWAs studies, including genetic variants in APOE, CLU, ABCA7, PICALM, BIN1, SORL1, PTK2B, EPHA1, CD33, CD2AP, MS4A6A, HLA-DRB5/HLA-DRB1, TREM1, TREM2, and TREML2 genes (Lambert, 2013; Replogle et al., 2015) which are assumed to be associated with AD through different pathways (Replogle et al., 2015; Giri et al., 2016). In particular, we focused on the potential associations of candidate SNPs frequency with the most common MRI markers associated with CAA in the lobar ICH cohort.

Furthermore, because of the particularly important role of ApoE and ApoJ in cerebral β-amyloidosis, we analyzed the association of the levels of total plasma ApoE and ApoJ proteins, as well as their content in different circulating lipoproteins, with the genetic variants in APOE and CLU genes related to AD. Finally, with the aim to improve our understanding of ApoE and ApoJ in cerebral β-amyloidosis, the associations between ApoE and ApoJ levels in plasma and lipoproteins with the most common MRI markers associated with CAA were also analyzed.

## 2. Materials and methods

### 2.1. Study population

The population studied in this cohort consisted of 126 participants who had suffered at least one lobar ICH and presented a clinical suspicion of CAA. Patients were recruited during a follow-up visit in 11 different Spanish centers (Bonaterra-Pastra et al., 2021; Marazuela et al., 2021). All patients were >55 years old and those presenting with symptomatic deep ICHs or treated with anticoagulant therapy were excluded.

The data obtained from the cohort included the recruitment date, date of stroke events, demographic characteristics (sex and age), relevant vascular risk factors [hypertension (HT), diabetes mellitus (DM), and dyslipidemia (DL)], medication, and cognitive status. Hypertension was defined as systolic BP ≥140 mmHg, diastolic BP ≥90 mmHg, or use of antihypertensive medication. DM was defined as fasting glucose levels >7 mmol/L or the use of antidiabetic drugs or insulin. Dyslipidemia was based on total cholesterol levels above 6.20 mmol/L or the use of lipid-lowering medication. Patients that presented mild cognitive impairment or dementia were all considered as cognitive impaired patients. The study was approved by the Clinical Investigation Ethical Committee of the Vall d’Hebron University Hospital, Barcelona, Spain (PR(AG)326/2014) and had the approval of the Ethical Committees of all the participating centers. The study was conducted in accordance with the Helsinki Declaration.

### 2.2. Magnetic resonance imaging evaluation

Brain MRI was acquired using a 1.5-T whole-body scanner for most patients (*n* = 118, 96%) for diagnostic and clinical purposes in each center at 6.70 ± 16.79 months from the last lobar ICH. The following sequences were obtained: axial T2-weighted turbo spin-echo, axial T1-weighted spin-echo, axial T2-weighted turbo fluid-attenuated inversion recovery (FLAIR), and axial T2*-weighted echo-planar gradient-echo sequence. All MRI images were evaluated in Vall d’Hebron University Hospital (VHUH) by the same neuroradiologist, who was blinded to clinical and biological information.

Intracerebral hemorrhages were defined as hypointense foci >5 mm in diameter on the T2*-weighted images, and the number and location were recorded. cSS was defined as the presence of thin hypointensity on T2*-weighted in a curvilinear pattern following the gyral cortical surface and was evaluated and classified as focal (restricted to ≤3 sulci) or disseminated (≥4 sulci) according to its distribution and severity as previously described (Linn et al., 2010). cSS contiguous or potentially anatomically connected with any lobar ICH were not included in the aforementioned categories. Perivascular spaces (PVS) also known as Virchow–Robin spaces were evaluated on axial T2-weighted images. Enlarged perivascular spaces (EPVS) in the basal ganglia (BG) or centrum semiovale (CSO) were rated using a previously described validated 4-point visual rating scale (0 = no EPVS, 1 ≤ 10 EPVS, 2 = 11–20 EPVS, 3 = 21–40 EPVS, and 4 > 40 EPVS) (Wardlaw et al., 2013). EPVS were also classified as low (≤20 EPVS) or high (≥21 EPVS) degree. The presence of CMB (diameter <5 mm), together with their number and location, was recorded following the Brain Observer MicroBleed Scale (BOMBS) (Cordonnier et al., 2009). Depending on the number of lobar microbleeds, patients were classified as having less than 5 lobar CMB or more than 5 (Lobar CMB >5). WMH were defined as hyperintense signal lesions in T2-FLAIR or T2-weighted images and were classified as deep or periventricular depending on their location. Deep and periventricular WMH were assessed according to the four-point Fazekas rating scale (Fazekas et al., 1987). For periventricular WMH: 0 = absence, 1 = “caps” or pencil-thin lining, 2 = smooth “halo,” 3 = irregular WMH extending into the deep white matter; and for deep WMH: 0 = absence, 1 = punctate foci, 2 = beginning confluence of foci, 3 = large confluent areas. We further classified WMH burden as mild (Fazekas: 0, 1) or severe (Fazekas: 2, 3). The WMH score was evaluated in the hemisphere not affected by hemorrhage, except in cases when both hemispheres were involved. Global brain atrophy was visually assessed and classified as mild, moderate or severe. The MRI analysis allowed the classification of cases according to Boston criteria 1.5 (Linn et al., 2010); 106 patients accomplished a CAA diagnosis, whereas 13 patients presented mixed-pathology (presenting lobar and deep microbleeds), 7 patients were excluded from the Boston criteria classification because of missing imaging information, and 1 patient was diagnosed as probable CAA with supporting pathology. In addition, all patients were classified according to the CAA-SVD burden score (Charidimou et al., 2016). It ranges from 0 to 6 points and it is based on the presence and grade of MRI markers associated with CAA including CMBs, cSS, CSO-EPVS, and WMH. In our study, the CAA-SVD burden was classified as low (≤3) or high degree (≥4).

### 2.3. Blood collection

Ten milliliters of blood in EDTA-containing Vacutainer tubes (Becton Dickinson, Franklin Lakes, NJ, USA) were collected from each participant at a follow-up visit. Blood was centrifuged at 4°C for 15 min at 2,500 rpm and plasma was immediately aliquoted and frozen at −80°C.

### 2.4. Genetic determinations

Single nucleotide polymorphisms (SNPs) candidates were genotyped from blood using SNPlex™ at the Spanish National Genotyping Centre (CEGEN) and included rs6656401 and rs6701713 of CR1, rs4311 of ACE, rs11136000, rs7012010, rs9331896, and rs9331888 of CLU, rs4147929 and rs3764650 of ABCA7, rs3851179 and rs10792832 of PICALM, rs744373 and rs6733839 of BIN1 gene, rs11767557 and rs11771145 of EPHA1, rs3865444 of CD33, rs9349407 and rs10948363 of CD2AP, rs11218343 of SORL1, rs9271192 of HLA-DRB5-DRB1 region, rs28834970 of PTK2B, rs6910730 of TREM1, rs75932628 of TREM2, and rs3747742 of TREML2, and rs4938933 and rs983392 of MS4A6A and rs429358 and rs7412 of the APOE gene to determine APOEε genotype. The minor allele frequency (MAF) of the SNPs analyzed in our cohort are reported in Supplementary Table 1. The information about MAF in the global population was obtained from 1,000 Genome Project phase 3 (Auton et al., 2015).

### 2.5. Plasma ApoE and ApoJ levels

Apolipoproteins levels determination in plasma and in lipoprotein fractions was done in a representative subcohort of 60 lobar ICH patients with clinical suspicion of CAA randomly selected. The lipoprotein fractions determined were very low-density lipoproteins (VLDL), low-density lipoproteins (LDL) and high-density lipoproteins (HDL). Blood samples from this group were obtained 10.42 ± 16.77 months after the last ICH and processed as previously explained. The standard lipid profile was quantified in the Clinical Biochemistry Unit of the VHUH using a direct HDL-cholesterol method (HDL-C plus) or by ultracentrifugation when the triglycerides concentration was higher than 3 mmol/L, in agreement with the National Cholesterol Education Program (National Cholesterol Education Program [NCEP] Expert Panel on Detection, Evaluation, and Treatment of High Blood Cholesterol in Adults [Adult Treatment Panel III]., 2002). These determinations were performed in an AU 5800 autoanalyzer (Beckman Coulter, Pasadena, CA, USA) using reagents from Beckman Coulter. To study the apolipoprotein content in the lipoprotein fractions, lipoproteins were isolated by flotation sequential ultracentrifugation, using 1 ml of plasma, according to density: VLDL (1.006–1.019 g/ml), LDL (1.019–1.063 g/ml), and HDL (1.063–1.210 g/ml) in the Research Institute of the Hospital de Sant Pau. Density was adjusted by adding the appropriate quantity of KBr, according to the equation of Radding and Steinberg (1960). Each sequential step consisted of ultracentrifugation at the aforementioned density at 100,000 *g* for 24 h, at 4°C, using 3.2 ml open-top Thickwall Polycarbonate tubes (Ref. No. 362305, Beckman Coulter). VLDL was collected in 400 μl, and LDL and HDL in 600 μl fractions of the upper phase. ApoE determination was determined in a Cobas 6000/c501 autoanalyzer using reagents from Kamiya Biomedicals (Cat. No. KAI-007, Seattle, WA, USA), using the parameters for automatic analyzer provided by the manufacturer. ApoE in both plasma and lipoprotein fractions were measured undiluted. ApoJ quantification, in plasma and lipoproteins, was analyzed by ELISA (#3713-1HP-1, Mabtech, Stockholm, Sweden) at VHIR. Before ELISA quantification, lipoprotein samples were diluted to the same cholesterol concentration (VLDL: 0.1 mmol cholesterol/L; LDL: 0.4 mmol cholesterol/L; HDL: 0.3 mmol cholesterol/L). Samples with null values for a particular quantification were discarded from such analysis.

### 2.6. Statistical analysis

To study allelic associations, each SNP was dissected into its two alleles to perform an allelic test. The association of all the SNPs and demographic variables with CAA neuroimaging features was studied by raw analysis using contingency tables and a χ^2^ test using the Pearson or Fisher *p*-value as needed. To adjust the results, a forward LR binary logistic regression using sex, age, and the significant SNPs for each radiological feature was assessed. The odds ratios (ORs) and 95% confidence intervals (CIs) for the effect on radiological characteristics were estimated using binary logistic regression analysis. This regression was performed as a common dominant model using SNPs as binary categorical variables [not having the minor allele (MA) vs. having the minor allele] or as an additive model (comparing the three different genotypes). For the binary logistic regression of the SVD burden, a simplified score of low-high SVD burden score was used. In addition, *p*-values were adjusted for multiple corrections by Benjamini–Hochberg (BH) false discovery rate (FDR). The distribution of the continuous variables was tested using the Kolmogorov–Smirnov test. If the distribution was normal, an independent samples *t*-test was performed, and if the distribution was not normal an independent samples Mann–Whitney U test. When analyzing the association between apolipoprotein levels and age or ordinal variables with more than 2 categories, Spearman correlations were performed. Data are expressed as the mean ± standard deviation (SD) for normal distributions or as the median [interquartile range] for non-normal distributions. To adjust our results, backward linear regressions were performed for ApoJ and ApoE circulating levels using the significant variables for each apolipoprotein fraction and adjusting by sex and age. The slope of the regression (*B*) with a 95% CI was estimated by linear regressions. A *p*-value below 0.05 was considered statistically significant. Statistical analyses were conducted using SPSS Statistics version 21 (IBM Corporation, Armonk, NY, USA) and R (R Foundation for Statistical Computing, Vienna, Austria).

## 3. Results

The demographic characteristics of the study population are displayed in Table 1. All patients presented at least one lobar ICH, 30.2% of patients presented 2 or more lobar ICH and 50.4% presented cognitive impairment. The most common MRI features previously associated with CAA were analyzed and resulted well represented in our cohort (Table 2). From them, 42.4% of patients presented cSS, 70% of whom displayed disseminated cSS. Almost all patients (92.4%) presented MRI-visible EPVS; 97.25% of them in the BG, mostly with low degree (80%), whereas 80.7% presented EPVS in the CSO, among them 48.9% with high degree. Also, 61.9% of the population presented CMB. Among them, most of the patients showed lobar CMBs (94.52%), including 36.23% that presented more than 20 lobar CMBs. Also, nearly the whole cohort (91.5%) presented WMH, both periventricular (89.8%) and deep (90.7%). Additionally, 37.3% of patients presented brain atrophy. A total of 11.1% of the cohort, and 10% of the subcohort for biomarker analysis in plasma, presented mixed pathology.

**TABLE 1:** Demographic and clinical characteristics.

Lobar ICH (*n* = 126)	
Age, years	76.18 ± 7.11
Sex (females)	68 (54%)
HT	73 (60.3%)
DM	18 (15.5%)
DL	41 (36.9%)
Cognitive impairment	58 (50.4%)
Corticosubcortical ICH	126 (100%)
≥2	38 (30.2%)
≥3	13 (10.3%)

Data are expressed as n (%). ICH, intracerebral hemorrhage; HT, hypertension; DM, diabetes mellitus; DL, dyslipidemia.

**TABLE 2:** Magnetic resonance imaging characteristics of a lobar ICH cohort with clinical suspicion of CAA (*N* = 118).

cSS	50 (42.4%)
Focal	15 (30%)
Disseminated	35 (70%)
**EPVS**	**109 (92.4%)**
BG-EPVS	106 (97.25%)
Low degree (1–20)	85 (80.2%)
High degree (21 to >40)	21 (19.8%)
CSO-EPVS	88 (80.7%)
Low degree (1–20)	45 (51.1%)
High degree (21 to >40)	43 (48.9%)
**CMB**	**73 (61.9%)**
Lobar CMB	69 (94.52%)
1–5	25 (36.23%)
6–10	12 (17.39%)
11–20	7 (10.14%)
>20	25 (36.23%)
Deep CMB	14 (19.18%)
1–5	12 (85.7%)
6–10	1 (7.1%)
11–20	1 (7.1%)
>20	0 (0%)
Cerebellum CMB	15 (20.5%)
1–5	12 (80%)
6–10	3 (20%)
11–20	0 (0%)
>20	0 (0%)
**WMH**	**108 (91.5%)**
Periventricular	97 (89.8%)
Caps or pencil/thin lining (1)	24 (24.7%)
Smooth halo or irregular periventricular (2–3)	73 (75.3%)
Deep	98 (90.7%)
Punctate foci (1)	43 (43.9%)
Beginning or large confluent areas (2–3)	55 (56.1%)
**Cortico-subcortical atrophy**	**44 (37.3%)**
Mild	29 (65.9%)
Moderate	13 (29.5%)
Severe	2 (4.5%)

The percentages displayed are within each category. Data are expressed as *n* (%). cSS, cortical superficial siderosis; EPVS, enlarged perivascular spaces; BG, basal ganglia; CSO, centrum semiovale; CMB, cerebral microbleeds; WMH, white matter hyperintensity.

**FIGURE 1:**
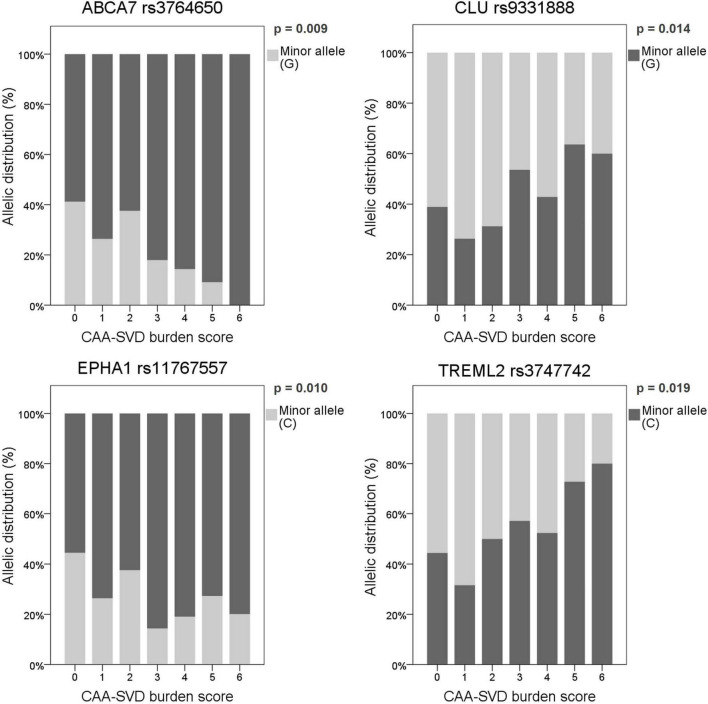
Allelic distribution of the SNPs associated with the CAA-SVD burden score using the common dominant model. The genetic risk factor (presenting or not the minor allele) associated with a higher score is represented in dark gray, whereas the protective factor is represented in light gray. Allelic distribution is expressed as a percentage in each category of the score (0–6).

The main aim of this study was to evaluate whether genetic variants described as AD risk factors were also related to CAA, especially focusing on imaging features associated with this pathology in patients with lobar ICH. To do so, associations between the frequency of the candidate SNPs and MRI features and demographic characteristics were considered through allelic univariant analysis (Supplementary Table 2). After applying a binary logistic regression adjusted by age and sex, only the candidate SNPs that remained significantly associated with specific MRI features are presented in Table 3. We detected that several CLU SNPs were associated with MRI features associated with CAA, such as the presence of cSS, CSO-EPVS, lobar CMB, and both deep and periventricular WMH. In addition, specific SNPs in ABCA7 (rs3764650; *p* = 0.009), CLU (rs9331888; *p* = 0.014), EPHA1 (rs11767557; *p* = 0.010), and TREML2 (rs3747742; *p* = 0.019) were independently associated with the CAA-SVD burden score when the common dominant model was applied (Figure 1). Moreover, the variants in ABCA7 (rs3764650; *p* = 0.013), CLU (rs9331896; *p* = 0.018), EPHA1 (rs11767557; *p* = 0.006), and TREML2 (rs3747742; *p* = 0.033) were also independently associated with the CAA-SVD burden score when the additive model was applied (Supplementary Figure 1). The minor alleles in the specific SNPs of ABCA7 [rs3764650 (G)], EPHA1 [rs11767557 (C)], and CLU [rs9331896 (C)] resulted to act as potential protective factors as their frequency significantly decreased at higher CAA-SVD score. On the other hand, the minor alleles in the specific SNPs of CLU [rs9331888 (G)] and TREML2 [rs3747742 (C)] were considered a risk factor, since their frequency was significantly correlated with a higher CAA-SVD score (Table 3). Interestingly, carriers of APOEε2 showed a statistical tendency to present a higher CAA-SVD burden score (Supplementary Table 2), however, the association was lost after the logistic regression.

**TABLE 3:** Binary logistic regression for CAA MRI markers with SNPs minor alleles adjusting by sex and age.

	Genetic and clinical variables	Common dominant model			Additive model		
		OR (95% CI)	*p*-Value	BH *p*-Value	OR (95% CI)	*p*-Value	BH *p*-Value
cSS	CLU rs9331888 (G allele, MA)	2.490 (1.175–5.278)	0.017	0.029	1.889 (1.033–3.454)	0.039	0.041
cSS extent	CD2AP rs10948363 (G allele, MA)	0.188 (0.042–0.836)	0.028	0.040	–	–	–
	CD2AP rs9349407 (C allele, MA)	–	–	–	0.150 (0.039–0.581)	0.006	0.024
	CLU rs11136000 (T allele, MA)	6.722 (1.500–30.127)	0.013	0.028	8.264 (1.698–40.000)	0.009	0.024
CSO-EPVS	–	–	–	–	–	–	–
High degree CSO-EPVS	CLU rs7012010 (C allele, MA)	2.370 (1.073–5.237)	0.033	0.043	1.935 (1.082–3.462)	0.026	0.033
BG-EPVS	–	–	–	–	–	–	–
High degree BG-EPVS	CD33 rs3865444 (A allele, MA)	–	–	–	3.333 (1.437–7.752)	0.005	0.024
Lobar CMB	ABCA7 rs3764650 (G allele, MA)	0.313 (0.126–0.778)	0.012	0.028	0.290 (0.115–0.728)	0.008	0.024
	CLU rs7012010 (C allele, MA)	2.185 (1.006–4.749)	0.048	0.048	1.853 (1.016–3.378)	0.044	0.044
Lobar CMB >5	ABCA7 rs3764650 (G allele, MA)	0.323 (0.110–0.945)	0.039	0.044	–	–	–
	CLU rs11136000 (T allele, MA)	–	–	–	0.503 (0.278–0.911)	0.023	0.031
	TREML2 rs3747742 (C allele, MA)	2.306 (1.44–5.092)	0.039	0.044	1.918 (1.095–3.362)	0.023	0.031
High deep WMH burden	CR1 rs6701713 (A allele, MA)	0.420 (0.177–0.994)	0.048	0.048	0.419 (0.191–0.919)	0.030	0.036
	CLU rs9331888 (G allele, MA)	3.054 (1.404–6.642)	0.005	0.028	2.351 (1.234–4.478)	0.009	0.024
High periventricular WMH burden	CLU rs9331888 (G allele, MA)	2.673 (1.211–5.897)	0.015	0.028	–	–	–
	CLU rs9331896 (C allele, MA)	–	–	–	0.478 (0.270–0.848)	0.012	0.027
Atrophy	BIN1 rs6733839 (T allele, MA)	0.307 (0.132–0.715)	0.002	0.028	0.457 (0.237–0.881)	0.019	0.031
	CD2AP rs10948363 (G allele, MA)	0.257 (0.111–0.595)	0.006	0.028	0.359 (0.170–0.755)	0.007	0.024
	CLU rs7012010 (C allele, MA)	–	–	–	2.101 (1.125–3.924)	0.020	0.031
CAA-small vessel disease burden score	ABCA7 rs3764650 (G allele, MA)	0.261 (0.096–0.711)	0.009	0.028	0.284 (0.105–0.764)	0.013	0.027
	CLU rs9331896 (C allele, MA)	–	–	–	0.470 (0.251–0.879)	0.018	0.031
	CLU rs9331888 (G allele, MA)	2.921 (1.246–6.851)	0.014	0.028	–	–	–
	EPHA1 rs11767557 (C allele, MA)	0.285 (0.109–0.744)	0.010	0.028	0.288 (0.118–0.698)	0.006	0.024
	TREML2 rs3747742 (C allele, MA)	2.787 (1.184–6.650)	0.019	0.029	1.963 (1.055–3.652)	0.033	0.037

BH *p*-value, adjusted Benjamini–Hochberg *p*-value; cSS, cortical superficial siderosis; EPVS, enlarged perivascular spaces; CSO, centrum semiovale; CMB, cerebral microbleeds; WMH, white matter hyperintensity; OR, odds ratio; CI, confidence interval; MA, minor allele.

**FIGURE 2:**
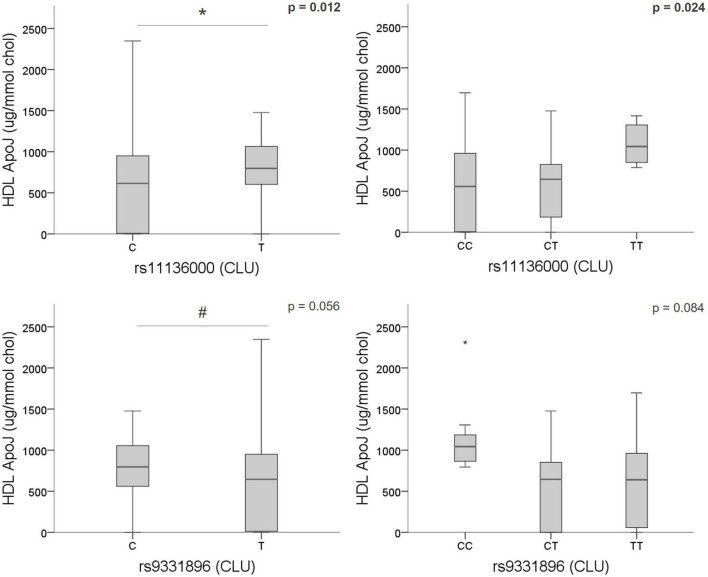
Association of CLU SNPs [rs1136000 (T), rs9331896 (C)] with ApoJ content in HDL. Boxplot representation of ApoJ content in HDL expressed as μg of ApoJ per mmol of cholesterol in HDL according to CLU SNPs alleles. *p < 0.05; ^#^*p* < 0.1.

From these results and considering the known role of ApoJ/CLU in cerebral β-amyloidosis (Howlett et al., 2013; Beeg et al., 2016), we next focused on the study of the potential genotypic association of the CLU SNPs (rs11136000, rs7012010, rs9331888, and rs9331896) with ApoJ levels in plasma, as well as its distribution among lipoproteins in samples from a subgroup of 60 patients with clinical suspicion of CAA. First, none of the demographic characteristics and main vascular risk factors evaluated were related with ApoJ levels in plasma or its content in VLDL, LDL, or HDL lipoproteins (Supplementary Table 3). However, the variant rs1113600 was significantly associated with ApoJ content in HDL [T allele (MA) = 795.87 [586.48–1,065.01] μg/mmol cholesterol (chol) vs. C allele = 614.68 [0–950.47] μg/mmol chol; *p* = 0.012], whereas the association between rs9331896 and HDL-ApoJ content showed a statistical tendency [C allele (MA) = 795.87 [549.54–1,065.01] μg/mmol chol vs. T allele = 644.44 [13.22–961.01] μg/mmol chol; *p* = 0.056]. In both cases, the minor allele [rs1113600 (T) and rs9331896 (C)] was associated with higher ApoJ content in HDL (Figure 2). No other associations were detected between CLU SNPs and circulating ApoJ levels (Supplementary Table 4). These associations remained significant when including only patients with blood samples obtained >90 days after ICH (*N* = 40) (Supplementary Table 5).

**FIGURE 3:**
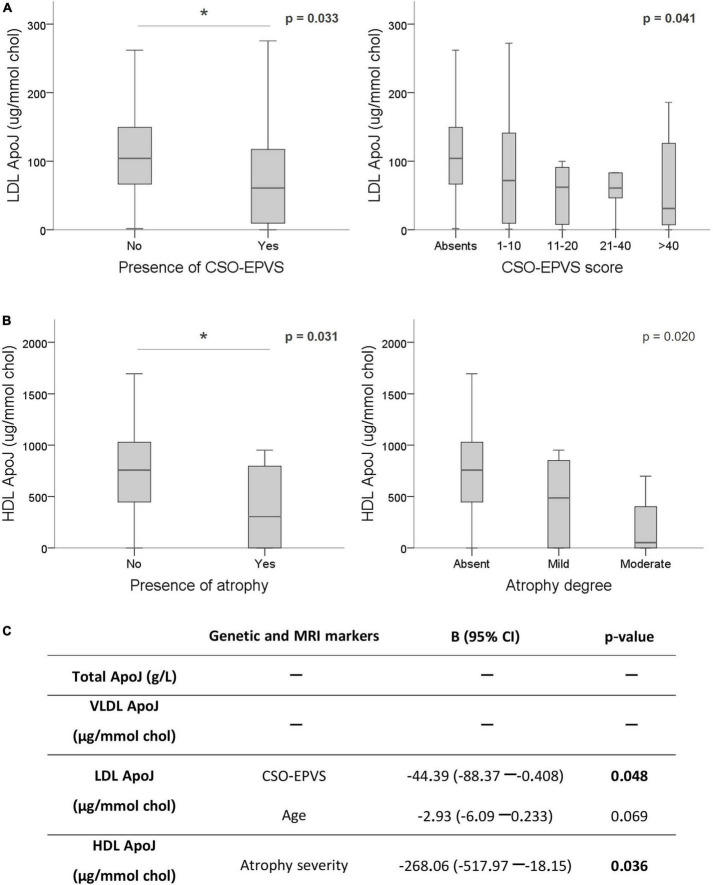
Association of ApoJ levels with MRI features associated with CAA. ApoJ levels are expressed as μg of ApoJ per mmol of cholesterol in each lipoprotein. **(A)** Boxplot representation of ApoJ levels in LDL according to EPVS in centrum semiovale (CSO). **(B)** ApoJ levels in HDL according to the presence and severity of cortico-subcortical atrophy. **(C)** Linear regression for circulating ApoJ levels using the significant variables for each lipoprotein fraction (CLU SNPs and MRI markers) and adjusting by sex and age. *p < 0.05.

On the other hand, whereas total plasma ApoJ levels resulted not linked to CAA-MRI markers, several associations were identified regarding ApoJ distribution among lipoproteins (Supplementary Table 2). Remarkably, lower ApoJ content in LDL was significantly associated with the presence of CSO-EPVS (absence = 104.06 [66.49–149.33] μg/mmol chol vs. presence = 60.78 [9.57–117.16] μg/mmol chol; *p* = 0.033), and with the corresponding EPVS rating scale (*p* = 0.041) (Figure 3A). In addition, lower ApoJ content in HDL was significantly associated with corticosubcortical atrophy (absence = 756.79 [466.97–1,028.20] μg/mmol chol vs. presence = 304.47 [0.00–795.87] μg/mmol chol; *p* = 0.031) and its severity (*p* = 0.020) (Figure 3B). After applying a linear regression adjusting by age and sex to underscore those variables independently associated with the protein circulating levels, we only found that certain MRI features remained significantly associated with ApoJ distribution in lipoproteins whereas the association with CLU SNPs lost signification. On the other hand, CSO-EPVS remained associated with circulating LDL ApoJ content and corticosubcortical atrophy remained associated with HDL ApoJ, independently of CLU SNPs (Figure 3C). If only chronic samples (>90 days after ICH) were analyzed, no associations were found, probably due to a reduced sample size (*n* = 40).

We next explored the potential alteration of the circulating ApoE levels in the lobar ICH cohort. Although none of the demographic characteristics or clinical variables analyzed were associated with ApoE concentration in plasma or with its distribution among lipoproteins (Supplementary Table 6), we found significantly higher levels of total plasma ApoE in patients carrying the ε2 allele (non-APOEε2 carriers = 43.16 ± 14.02 mg/L vs. APOEε2 carriers = 67.04 ± 21.56 mg/L; *p* < 0.001) and significantly lower total plasma ApoE levels associated with the presence of the ε4 allele (non-APOEε4 carriers = 48.59 ± 17.17 mg/L vs. APOEε4 carriers = 36.82 ± 11.73 mg/L; *p* = 0.024) (Supplementary Table 7), as previously described (Montañola et al., 2016; Marais, 2019). Furthermore, the APOEε2 genotype was also significantly associated with higher levels of ApoE in LDL (non-APOEε2 carriers = 35.07 [17.64–51.61] μmol/mol chol vs. APOEε2 carriers = 67.48 [58.79–171.32] μmol/mol chol; *p* = 0.001) (Supplementary Table 7). The associations between APOE genotype and ApoE levels in plasma and lipoproteins remained significant when analyzing the subcohort of patients with blood samples obtained chronically (>90 days after ICH) (Supplementary Table 8).

**FIGURE 4:**
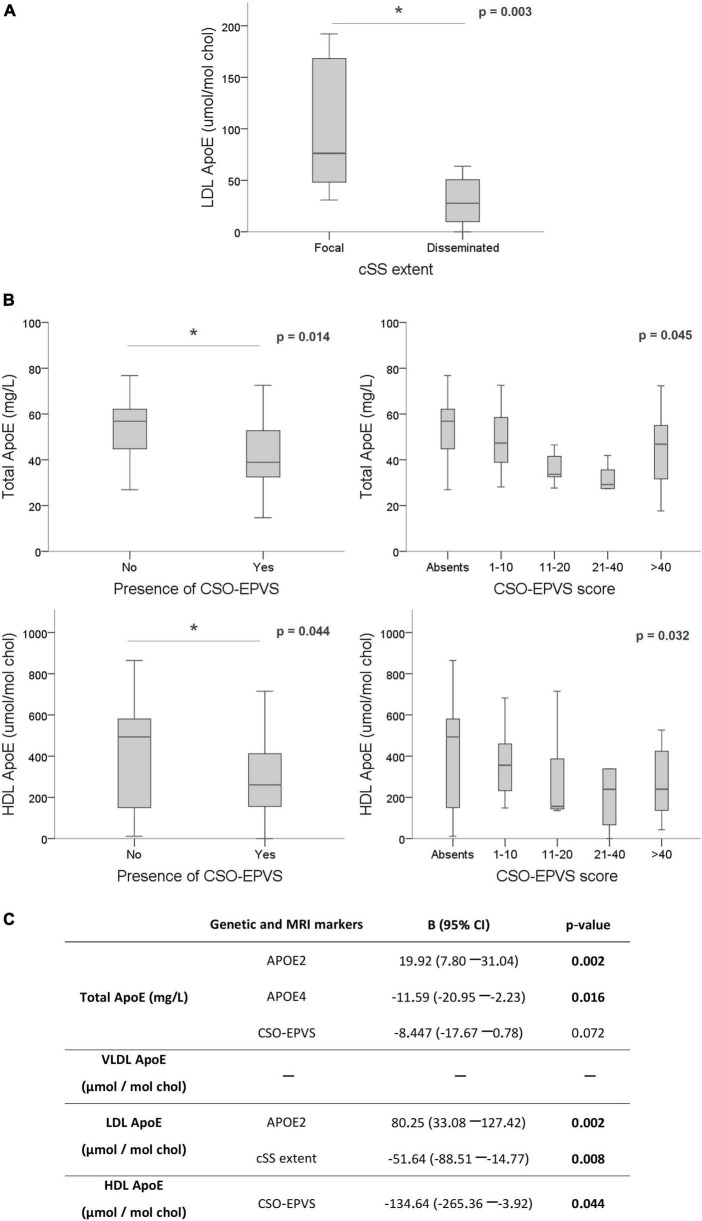
Association of ApoE levels with MRI features. ApoE levels are expressed as mg/L in plasma and μmol of ApoE per mol of cholesterol in each lipoprotein. **(A)** Boxplot distribution of ApoE levels in LDL according to cortical superficial siderosis severity and distribution. **(B)** Total and HDL ApoE levels according to EPVS in centrum semiovale (CSO). **(C)** Linear regression for circulating ApoE levels using the significant variables for each lipoprotein fraction (APOE genotypes and MRI markers) and adjusting by sex and age. *p < 0.05.

Interestingly, when analyzing the possible associations between circulating ApoE levels and the presence of the different MRI markers, we detected lower content of ApoE in LDL significantly associated with a higher extent of cSS (focal cSS = 76.19 [48.16–168.08] vs. disseminated cSS = 27.73 [9.85–60.51] μmol/mol chol; *p* = 0.003) (Figure 4A). This association was maintained when chronic plasma samples (>90 days after ICH) were exclusively analyzed (focal cSS = 124.93 [58.22–176.51] vs. disseminated cSS = 27.73 [11.32–60.30] μmol/mol chol; *p* = 0.010). Also, as observed with the ApoJ protein study, we found that ApoE content in lipoproteins was associated with the presence of visible CSO-EPVS and their degree score. Lower total and HDL ApoE levels were significantly correlated with the presence of CSO-EPVS (absence = 55.61 ± 21.15 mg/L and 441.36 ± 267.94 μmol/mol chol vs. presence = 42.92 ± 14.48 mg/L and 306.72 ± 192.85 μmol/mol chol; *p* = 0.014, and *p* = 0.044, respectively) and with the corresponding degree score (*p* = 0.045 and *p* = 0.032, respectively) (Figure 4B). Association between ApoE total and HDL levels with CSO-EPVS presence and severity remained significant for chronically obtained samples (>90 days after ICH) (absence = 57.10 [50.45–69.45] mg/L and 548.65 ± 229.02 μmol/mol chol vs. presence = 38.90 [32.90–49.55] mg/L and 301.62 ± 188.00 μmol/mol chol; *p* = 0.004 and *p* = 0.002, respectively; degree score *p* = 0.001 and *p* = 0.001, respectively). After a linear regression adjustment considering age and sex to underscore those variables independently associated with ApoE circulating levels, we found that APOEε2 and ε4 genotypes resulted independently associated with total plasma protein levels. In addition, APOEε2 genotype and cSS severity maintained the independent association with LDL ApoE levels. And finally, the presence of visible CSO-EPVS remained significantly associated with HDL ApoE levels (Figure 4C).

## 4. Discussion

Detecting the cerebrovascular Aβ pathology is a crucial endpoint with diagnostic value and potential for monitoring CAA progression. In recent years, the development of positron emission tomography (PET) radiotracers for Aβ imaging (Farid et al., 2017; Chang et al., 2021) and cerebrospinal fluid (CSF) biomarkers (Kuiperij et al., 2020) have been an intensive focus of research in the CAA field, although high costs and the invasiveness of these tests make them still limited for the clinical practice. The evolution of the vascular pathology could be potentially monitored through the progression of SVD neuroimaging markers strongly associated with CAA pathology, although most of these features have little specificity (Chojdak-Łukasiewicz et al., 2021). Furthermore, although these MRI markers reflect distinct pathophysiologic aspects of the disease, biological mechanisms underlying their occurrence and topography are still under investigation. In this regard, the hypothesis of the present study was that candidate genes previously associated with different pathological aspects in AD could also be related, in accordance or in opposition, to the deposition of Aβ in blood vessels and may shed light on the biological impact of the MRI features commonly found in CAA patients. For this purpose, we tested the MAF of candidate AD-linked SNPs identified in previous GWAS (Lambert, 2013; Replogle et al., 2015) in a multicenter cohort of patients that suffered a lobar ICH with a clinical suspicion of CAA.

Initially, we found that the frequency of minor alleles in certain candidate genetic variants in the ICH-CAA cohort did not follow the same direction as the results previously described for AD pathology. This phenomenon is well established for the presence of the APOEε2 allele, which is a protective genetic variant for AD, whereas it is associated with a higher hemorrhagic load in CAA (Lambert, 2013; Greenberg et al., 2020). In this sense, for instance, ABCA7 rs3764650 (G) has been described as a risk factor for AD, without a direct influence on CAA (Beecham et al., 2014). However, other ABCA7 SNPs have been associated with both CAA and small vessel ischemic disease (Mäkelä et al., 2018; Blumenau et al., 2020). In our cohort, we observed that ABCA7 SNP rs3764650 (G) acted as a protective factor regarding the presence and number of lobar CMBs and also resulted to be less frequent in those cases with a higher CAA-SVD burden score. Also, TREML2 rs3747742 (C), a protective factor for AD (Benitez et al., 2014), acted as a risk factor for the presence of a high number of lobar CMB (>5) and resulted more frequent when increasing the CAA-SVD burden score. Finally, EPHA1 rs11767557 (C) was significantly associated with lower punctuation in the neuroimaging CAA-SVD burden score. This SNP was previously related to a higher risk of AD; although other EPHA1 genetic polymorphisms have also been associated with small vessel ischemic disease (Wei et al., 2019; Blumenau et al., 2020). All these results add evidence to the complexity of pathophysiological processes that lead to the development of CAA or AD. ABCA7 has been previously associated with Aβ metabolism and neuritic plaque burden in AD (Shulman et al., 2013; Beecham et al., 2014; Mäkelä et al., 2018). Furthermore, ABCA7, in addition to TREML2 and EPHA1, has been proposed to influence Aβ clearance (Benitez et al., 2014; Karch and Goate, 2015; Mäkelä et al., 2018). Alterations in Aβ production and clearance may cause a redistribution of Aβ from parenchyma to vasculature, possibly causing a lower burden of neuritic plaques together with a major accumulation of Aβ in the cerebral vessels, which could explain how protective factors for AD could cause risk for CAA and vice versa. However, these genes are also involved in many biological mechanisms that may differently influence both pathologies, such as Tau pathology or BBB integrity maintenance (Obermeier et al., 2013; Shulman et al., 2013; Beecham et al., 2014; Benitez et al., 2014).

Interestingly, we observed that the four CLU SNPs studied were independently and significantly associated with several CAA neuroimaging markers. In more detail, rs7012010 (C) and rs9331888 (G) were previously widely described as risk markers for AD (Lambert et al., 2009; Yu and Tan, 2012; Tan et al., 2016; Foster et al., 2019). In the present study, we observed significant independent associations of rs7012010 (C) with the degree of CSO-EPVS and the presence of lobar CMB and brain atrophy; and rs9331888 (G) with the presence of cSS, WMH burden and a global higher CAA-SVD burden, as features presumably associated with a higher CAA severity in the lobar ICH population. On the contrary, rs11136000 (T) and rs9331896 (C) were previously described as protective factors for AD (Foster et al., 2019). In this sense, we observed rs9331896 (C) to be independently and significantly associated with a lower burden of periventricular WMH and CAA-SVD score and rs11136000 (T) with a reduced number of CMB, in line with a previous study of our group where rs11136000 (T) was associated with the absence of lobar CMBs (Montañola et al., 2016). Altogether, in the lobar-ICH cohort, the allelic frequencies of CLU SNPs rs9331896 (C) and rs9331888 (G) followed the pattern of potential risk/protective factors previously described in AD. A recent study found several different CLU haplotypes associated with lobar ICH, suggesting that different CLU SNPs are clearly involved in ICH and that different SNPs within the same gene could cause the same phenotypic endpoint (Sawyer et al., 2022). Regarding APOE genotype, the presence of ε2 and ε4 alleles had no impact on the common CAA MRI features, although APOEε2 carriers presented a statistical tendency to higher CAA-SVD burden score. Previous studies found an association of APOEε4 with strictly lobar CMB (Shams et al., 2022) and APOEε2 with cSS (Charidimou et al., 2015) although these results have not been confirmed in other studies (Shoamanesh et al., 2014; Montañola et al., 2016; Shams et al., 2022).

Because we found a relevant association of different CLU SNPs with the presence of several MRI features associated with CAA, we further explored their potential linked with ApoJ peripheral levels. In fact, it has been extensively described that certain CLU SNPs may affect ApoJ expression and levels (Szymanski et al., 2011; Xing et al., 2012; Cai et al., 2016; Tan et al., 2016). None of the SNPs analyzed was associated with changes in ApoJ plasma concentration, but some associations with the distribution of ApoJ in lipoproteins were observed. In this sense, we detected that the rs11136000 (T) and rs9331896 (C) SNPs were specifically related with higher content of ApoJ in HDLs. Indeed, rs11136000 (T) has been described to increase cerebral ApoJ expression in AD patients and controls (Allen et al., 2012; Ling et al., 2012), although studies regarding its impact on plasma levels have found different results (Schürmann et al., 2011; Mullan et al., 2013). Therefore, we observed that only the AD-protective CLU SNPs [rs11136000 (T) and rs9331896 (C)] were associated with higher ApoJ content in HDL, the circulating lipoprotein with higher content of ApoJ, in an allelic-dependent manner.

On the other hand, we used the same strategy regarding associations between genetic APOE variants and ApoE plasma concentration and its distribution among lipoproteins. Unlike what is observed for ApoJ, total plasma levels of ApoE are modulated by the APOE alleles. In this sense, we detected that carrying the ε2 allele significantly increased total plasma ApoE levels, whereas ε4 significantly decreased its levels as has been extensively described in different cohorts (Montañola et al., 2016; Rasmussen, 2016). Regarding ApoE distribution among plasma lipoproteins, we observed that APOEε2 carriers presented also significantly increased ApoE content in LDL. It is important to consider that apart from altering ApoE levels, APOE genotype also influences ApoE structure and functionality, and thus, affects processes such as the clearance of parenchymal Aβ through LDL receptor-related protein 1 (LRP1) (Yin and Wang, 2018).

In order to understand the biological impact of the MRI markers analyzed, we studied potential associations with ApoJ and ApoE circulating levels in the CAA-ICH cohort. ApoE and ApoJ content in both HDL and LDL are negatively associated with several MRI features. Specifically, we found that lower ApoJ content in LDL and lower ApoE levels in plasma and HDL were associated with CSO-EPVS presence and degree. EPVS in CSO have been related to high Aβ burden in CAA and AD patients in post-mortem histopathological studies (Roher et al., 2003; van Veluw et al., 2016; Perosa et al., 2022), suggesting that these pathological space enlargements may be the consequence of the impairment in the perivascular drainage pathways (Hawkes et al., 2011; Arbel-Ornath et al., 2013; Perosa et al., 2022). Interestingly, in a previous study, we found ApoJ LDL content to be significantly elevated in AD patients compared to controls, but not in the ICH-CAA cohort (Bonaterra-Pastra et al., 2021), suggesting that the content of ApoJ in LDL could influence the Aβ traffic within the brain. Moreover, it has been previously described that ApoJ treatment could improve HDL functionality regarding atheroprotective, anti-oxidative and anti-inflammatory properties (Rivas-Urbina et al., 2020). Also, ApoE in HDL could act as a protective factor since AD patients present HDL with lower ApoE content (Pedrini et al., 2022), and in our previous study, we observed that subjects from an ICH-CAA cohort presented a higher ratio of ApoE/ApoC-III in HDL than controls which we hypothesized to be a defensive response against vascular Aβ deposition (Bonaterra-Pastra et al., 2021). Higher circulating ApoE levels are known to be atheroprotective, and ApoE-enriched HDL are able to reduce CAA in an *in vitro* model (Morton et al., 2019; Robert et al., 2020). Altogether we propose that higher content of ApoJ and ApoE in LDL and HDL may enhance the drainage of Aβ from brain vessels, displaying protective properties. Both ApoE and ApoJ bind Aβ and are established ligands for different receptors of the LDL receptor family such as LDL receptor, ApoE receptor, VLDL receptor, LRP1, and megalin. Therefore, lower content of ApoJ and ApoE in lipoproteins may be related with higher vascular Aβ deposition, which could be translated to a more prominent occurrence and severity of different CAA-SVD markers.

## 5. Conclusion

In summary, we observed several genetic variants previously associated with AD (SNPs on ABCA7, BIN1, CD2AP, CLU, CR1, EPHA1, and TREML2) to be associated with MRI markers of CAA. In more detail, the CLU SNPs studied were independently and significantly associated with several CAA markers and followed the same risk/protective direction as previously described for AD. We observed CLU SNPs and APOE genotype described as protective factors for AD to be associated with higher ApoJ and ApoE content in lipoproteins. Furthermore, ApoJ and ApoE distribution among lipoproteins was associated with CAA-MRI features, such as the presence of CSO-EPVS and their degree score. We suggest that higher ApoE and ApoJ levels in HDL and LDL might enhance atheroprotective and anti-inflammatory responses in cerebral amyloidosis. This study reinforces the relevance of peripheral lipid metabolism and cerebrovascular functionality in CAA and could open the path for new therapeutic strategies. However, the meaning of our findings regarding ApoJ and ApoE distribution in lipoproteins and their link to Aβ pathology needs further research and confirmation in larger cohorts. An important limitation of our study is that all the patients from our cohort presented a symptomatic ICH and some of the results observed could be due to pathological changes associated with this hemorrhagic phenotype. We tried to overcome this issue by collecting the blood samples of the participants for protein determinations in a non-acute phase of the disease to avoid the massive inflammatory cascade produced after the stroke. Also, as an observational study, we cannot confirm the causality of the differences observed in ApoJ and ApoE circulating levels. Finally, although we are in front of a multicenter study, the sample size is still small and larger studies with a higher number of patients should be conducted, especially regarding the genetic association’s analysis.

## Data availability statement

The raw data supporting the conclusions of this article will be made available by the authors, without undue reservation.

## Ethics statement

The studies involving human participants were reviewed and approved by the Clinical Investigation Ethical Committee of the Vall d’Hebron University Hospital, Barcelona, Spain (PR(AG)326/2014). The patients/participants provided their written informed consent to participate in this study.

## Author contributions

MH-G: conceptualization. AB-P, CV, AR, and SB: methodology. OP, DR-L, MF, ST, MM-Z, PC, RV, LL-H, JA, SP-S, AD-M, JF, GM, and JM: recruitment of patients. AB-P and MH-G: formal analysis. AB-P: writing—original draft preparation. MH-G and JS-Q: writing—review and editing, supervision, and funding acquisition. All authors read and agreed to the published version of the manuscript.
