# Supplementary material for: Association of candidate genetic variants and circulating levels of ApoE/ApoJ with common neuroimaging features of cerebral amyloid angiopathy
Source: Front Aging Neurosci. 2023 Apr 11;15:1134399. doi: 10.3389/fnagi.2023.1134399 (PMC10126235; doi:10.3389/fnagi.2023.1134399)
Supplement: Supplementary file 1 [file Table_1.DOCX]

Supplementary Material

Association of candidate genetic variants and circulating levels of ApoE/ApoJ with common neuroimaging features of Cerebral Amyloid Angiopathy

Anna Bonaterra-Pastra^1^, Sònia Benítez^2,16^, Olalla Pancorbo ^3^, David Rodríguez-Luna^3^, Carla Vert^4^, Alex Rovira^4^, M. Mar Freijo^5^, Silvia Tur^6^, Maite Martínez-Zabaleta^7^, Pere Cardona Portela^8^, Rocío Vera^9^, Lucia Lebrato-Hernández^10^, Juan F. Arenillas^11^, Soledad Pérez-Sánchez^12^, Ana Domínguez-Mayoral^12^, Joan Martí Fàbregas^13^, Gerard Mauri^14^, Joan Montaner^1,15^, Jose Luis Sánchez-Quesada^2,16^*, and Mar Hernández-Guillamon^1^*.

*** Correspondence:** M.H-G. mar.hernandez.guillamon@vhir.org; J.L.S-Q. [jsanchezq@santpau.cat](mailto:jsanchezq@santpau.cat)

**Supplemental Table 1.**

|  | Global Population * | Study Population |
| --- | --- | --- |
| ABCA7 rs3764650  (G allele, MAF) | 0.20 | 0.23 |
| ABCA7 rs4147929  (A allele, MAF) | 0.18 | 0.44 |
| ACE rs4311 (A allele, MAF) | 0.33 | 0.79 |
| BIN1 rs6733839 (T allele, MAF) | 0.40 | 0.58 |
| BIN1 rs744373 (G allele, MAF) | 0.36 | 0.46 |
| CD2AP rs10948363  (G allele, MAF) | 0.19 | 0.49 |
| CD2AP rs9349407  (C allele, MAF) | 0.19 | 0.49 |
| CD33 rs3865444 (A allele, MAF) | 0.21 | 0.47 |
| CLU rs11136000 (T allele, MAF) | 0.38 | 0.37 |
| CLU rs7012010 (C allele, MAF) | 0.33 | 0.55 |
| CLU rs9331888 (G allele, MAF) | 0.33 | 0.44 |
| CLU rs9331896 (C allele, MAF) | 0.38 | 0.63 |
| CR1 rs6656401 (A allele, MAF) | 0.07 | 0.29 |
| CR1 rs6701713 (A allele, MAF) | 0.25 | 0.31 |
| EPHA1 rs11767557  (C allele, MAF) | 0.20 | 0.28 |
| EPHA1 rs11771145  (A allele, MAF) | 0.43 | 0.51 |
| HLA-DRB5/HLA-DRB1 rs9271192 (C allele, MAF) | 0.24 | 0.41 |
| MS4A4A-MS4A6A rs4938933 (C allele, MAF) | 0.38 | 0.76 |
| MS4A4A-MS4A6A rs983392 (G allele, MAF) | 0.23 | 0.75 |
| PICALM rs10792832  (A allele, MAF) | 0.31 | 0.44 |
| PICALM rs3851179  (T allele, MAF) | 0.31 | 0.56 |
| PTK2B rs28834970  (C allele, MAF) | 0.32 | 0.64 |
| SORL1 rs11218343  (C allele, MAF) | 0.11 | 0.05 |
| TREM1 rs6910730  (G allele, MAF) | 0.27 | 0.26 |
| TREM2 rs75932628  (T allele, MAF) | < 0.01 | < 0.01 |
| TREML2 rs3747742  (C allele, MAF) | 0.31 | 0.52 |

**ST1.** Allelic frequency of minor alleles in Global Population and the Population studied. MAF: Minor allele frequency. Data are expressed as frequency per 1. *Global Population MAF was obtained from 1000 Genome Project phase 3 (Auton et al., 2015).

**Supplemental Table 2**

|  | cSS | cSS extent | CSO-EPVS | CSO-EPVS degree | BG-EPVS | BG-EPVS degree | Lobar CMB | Lobar CMB >5 | WMH deep burden | WMH PV burden | Atrophy | CAA-SVD burden score  (low vs. high) |
| --- | --- | --- | --- | --- | --- | --- | --- | --- | --- | --- | --- | --- |
| Sex | 0.635 | 0.758 | 0.111 | 0.876 | 0.16 | 0.144 | 0.584 | 0.473 | 0.252 | 0.078 | 0.473 | 0.753 |
| Age | 0.946 | 0.256 | 0.538 | 0.282 | 0.669 | 0.086 | 0.759 | 0.458 | 0.173 | 0.126 | 0.144 | 0.483 |
| HT | 0.972 | 0.228 | 0.311 | 0.678 | 1 | 0.691 | 0.723 | 0.565 | 0.739 | 0.276 | 0.989 | 0.629 |
| DM | 0.233 | 0.647 | 0.561 | 0.438 | 0.686 | 0.304 | 0.229 | 0.076 | 0.962 | 0.682 | 0.401 | 0.183 |
| DL | 1.000 | 0.822 | 0.895 | 0.827 | 0.764 | 0.322 | 0.218 | 0.300 | 0.441 | 0.524 | 0.703 | 0.591 |
| ABCA7 rs3764650  (G allele, MA) | 0.143 | 1.000 | 0.614 | 0.215 | 0.495 | 0.593 | **0.009** | **0.029** | 0.488 | 0.229 | 0.079 | **0.013** |
| ABCA7 rs4147929  (A allele, MA) | 0.630 | 0.133 | 0.663 | 0.502 | 0.653 | 0.789 | **0.013** | 0.079 | 0.992 | 0.227 | **0.017** | 0.132 |
| ACE rs4311 (T allele, MA) | 0.684 | 0.570 | 0.648 | 0.446 | 0.487 | 0.481 | 0.988 | 0.31 | 0.958 | 0.467 | 0.949 | 0.855 |
| BIN1 rs6733839 (T allele, MA) | 0.837 | 0.927 | 0.128 | 0.973 | 0.878 | 0.832 | 0.639 | 0.656 | 0.088 | 0.721 | **0.032** | 0.748 |
| BIN1 rs744373 (G allele, MA) | 0.328 | 0.641 | 0.201 | 0.425 | 0.523 | 0.447 | 0.462 | 0.565 | 0.146 | 0.421 | 0.117 | 0.964 |
| CD2AP rs10948363  (G allele, MA) | 0.180 | **0.008** | 0.622 | 0.416 | 0.503 | 0.585 | 0.710 | 0.329 | 0.430 | 0.813 | **0.013** | 0.597 |
| CD2AP rs9349407  (C allele, MA) | 0.180 | **0.008** | 0.622 | 0.416 | 0.503 | 0.585 | 0.710 | 0.329 | 0.430 | 0.813 | **0.013** | 0.597 |
| CD33 rs3865444 (A allele, MA) | 0.729 | 0.302 | 0.611 | **0.036** | 0.92 | **0.005** | 0.090 | 0.317 | 0.468 | 0.489 | 0.591 | 0.676 |
| CLU rs11136000 (T allele, MA) | **0.046** | **0.031** | 0.305 | 0.176 | 0.84 | 0.113 | 0.139 | **0.017** | 0.090 | **0.009** | 0.198 | **0.006** |
| CLU rs7012010 (C allele, MA) | 0.559 | 0.146 | 0.672 | **0.025** | 0.694 | 0.176 | **0.034** | 0.142 | 0.937 | 0.321 | **0.009** | 0.101 |
| CLU rs9331888 (G allele, MA) | **0.033** | 0.852 | 0.300 | 0.274 | 0.136 | 0.844 | 0.714 | 0.120 | **0.010** | **0.020** | 0.534 | **0.023** |
| CLU rs9331896 (C allele, MA) | **0.041** | **0.042** | 0.235 | 0.248 | 0.91 | 0.264 | 0.149 | **0.028** | 0.147 | **0.010** | 0.417 | **0.006** |
| CR1 rs6656401 (A allele, MA) | 0.331 | 0.601 | 0.260 | 0.315 | 0.216 | 0.153 | 0.527 | 0.373 | 0.290 | 0.990 | 0.612 | 0.786 |
| CR1 rs6701713 (A allele, MA) | 0.299 | 0.201 | 0.279 | 0.671 | 0.385 | 0.567 | 0.397 | 0.503 | **0.043** | 0.360 | 0.761 | 0.491 |
| EPHA1 rs11767557  (C allele, MA) | **0.043** | 0.272 | 0.784 | 0.881 | 0.544 | 0.98 | 0.308 | 0.305 | 0.072 | 0.124 | 0.520 | **0.012** |
| EPHA1 rs11771145  (A allele, MA) | 0.813 | 0.496 | 0.924 | 0.406 | 0.606 | 0.476 | 0.480 | 0.916 | 0.101 | 0.541 | 0.432 | 0.062 |
| HLA-DRB5/HLA-DRB1 rs9271192 (C allele, MA) | 0.663 | 0.691 | 0.796 | 0.721 | 0.653 | 0.789 | 0.469 | 0.292 | 0.992 | 0.728 | 0.411 | 0.533 |
| MS4A4A-MS4A6A rs4938933 (C allele, MA) | 0.491 | 0.598 | 0.992 | 0.572 | 0.936 | 0.844 | 0.748 | 0.784 | 0.739 | 0.582 | 0.784 | 0.496 |
| MS4A4A-MS4A6A rs983392 (G allele, MA) | 0.184 | 0.479 | 0.715 | 0.903 | 0.901 | 0.348 | 0.666 | 0.869 | 0.945 | 0.721 | 0.869 | 0.764 |
| PICALM rs10792832  (A allele, MA) | 0.366 | 0.532 | 0.392 | 0.706 | 0.737 | 0.863 | 0.764 | 0.922 | 0.318 | 0.778 | 0.179 | 0.809 |
| PICALM rs3851179  (T allele, MA) | 0.432 | 0.532 | 0.346 | 0.628 | 0.772 | 0.811 | 0.674 | 0.78 | 0.394 | 0.863 | 0.244 | 0.712 |
| PTK2B rs28834970  (C allele, MA) | 0.533 | 0.109 | 0.671 | 0.090 | 0.672 | 0.198 | 0.995 | 0.614 | 0.791 | 0.478 | 0.959 | 0.503 |
| SORL1 rs11218343  (C allele, MA) | 0.246 | 1.000 | 1.000 | 0.194 | 1 | 1 | 0.242 | 0.415 | **0.031** | **0.031** | 1.000 | 0.412 |
| TREM1 rs6910730  (G allele, MA) | 0.314 | 0.426 | 0.442 | 0.964 | 1 | 0.22 | 0.492 | 0.594 | 0.658 | 0.165 | 0.181 | 0.430 |
| TREM2 rs75932628  (T allele, MA) | 1.000 | - | 0.254 | 1.000 | 1 | 1 | 0.420 | 1 | 0.466 | 1.000 | 0.373 | 0.449 |
| TREML2 rs3747742  (C allele, MA) | 0.284 | 0.716 | 0.828 | 0.930 | 0.737 | 0.863 | **0.039** | **0.013** | 0.472 | 0.253 | 0.500 | **0.023** |
| APOEε2 | 0.306 | 0.713 | 0.359 | 0.862 | 0.446 | 0.205 | 0.725 | 0.933 | 0.286 | 0.320 | 0.561 | 0.097 |
| APOEε4 | 0.468 | 0.705 | 0.395 | 0.176 | 1 | 0.771 | 0.459 | 0.434 | 0.455 | 0.829 | 0.434 | 0.917 |
| Total ApoJ (g/L) | 0.282 | 0.571 | 0.888 | 0.620 | 0.608 | 0.820 | 0.711 | 0.953 | 0.364 | 0.432 | 0.107 | 0.858 |
| VLDL ApoJ (µg/mmol chol) | 0.815 | 1.000 | 0.064 | 0.712 | 0.388 | **0.033** | 0.367 | 0.302 | 0.624 | 0.664 | 0.827 | 0.269 |
| LDL ApoJ (µg/mmol chol) | 0.511 | 0.849 | **0.033** | 0.223 | 0.737 | **0.022** | 0.513 | 0.067 | 0.194 | 0.497 | 0.253 | 0.432 |
| HDL ApoJ (µg/mmol chol) | 0.615 | 0.285 | 0.104 | 0.181 | 0.374 | 0.306 | 0.419 | 0.648 | 0.244 | 0.484 | **0.031** | 0.872 |
| Total ApoE (mg/L) | 0.778 | 0.201 | **0.014** | 0.121 | 0.055 | 0.943 | 0.237 | 0.344 | 0.754 | 0.457 | 0.933 | 0.924 |
| VLDL ApoE  (µmol / mol chol) | 0.506 | 0.338 | 0.598 | 0.211 | 0.891 | 0.759 | 0.481 | 0.790 | 0.756 | 0.083 | 0.562 | 0.701 |
| LDL ApoE  (µmol / mol chol) | 0.833 | **0.003** | 0.408 | 0.178 | 0.334 | 0.631 | 0.407 | 0.403 | 0.816 | 0.689 | 0.756 | 0.785 |
| HDL ApoE  (µmol / mol chol) | 0.537 | 0.256 | **0.044** | 0.096 | 0.087 | 0.654 | 0.703 | 0.898 | 0.413 | 0.586 | 0.498 | 0.508 |

**ST2.** Association of SNPs, circulating ApoE and ApoJ levels, and demographic variables with CAA- MRI common features. The data displayed are unadjusted p-values. cSS: cortical Superficial Siderosis; distr.: distribution; EPVS: Enlarged Perivascular Spaces; BG: Basal Ganglia; CSO: Centrum Semiovale; BG: Basal Ganglia; CMB: Cerebral Microbleedings; WMH: White Matter Hyperintensity; PV: periventricular. MA: Minor Allele

**Supplemental Figure 1**

**
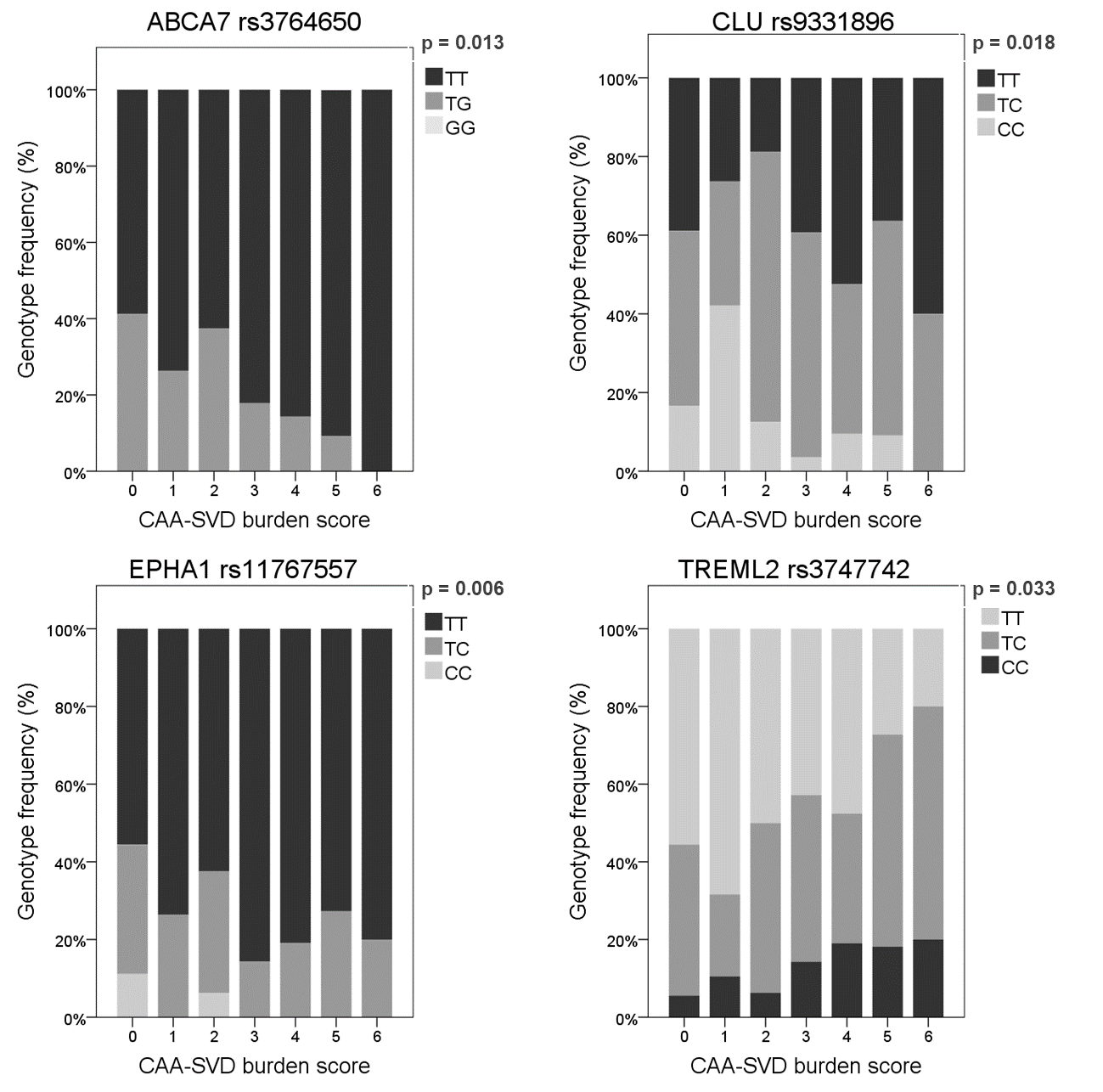
**

**SF1.** Genotype frequency of the SNPs associated with the CAA-SVD burden score using the additive model. The genetic risk factor associated with a higher score is represented in darker grey, whereas the protective factor is represented in lighter grey. Genotype frequency is expressed as a percentage in each category of the score (0-6).

**Supplemental Table 3**

|  | | **Total ApoJ (g/L)** N=59 | **VLDL ApoJ (µg/mmol chol)** N= 60 | **LDL ApoJ (µg/mmol chol)** N=60 | **HDL ApoJ  (µg/mmol chol)** N=60 |
| --- | --- | --- | --- | --- | --- |
| **Sex** | M | 0.183 ± 0.060 | 177.99 [113.78 – 217.36] | 60.78 [4.34 – 93.81] | 450.98 [0 – 881.95] |
|  | F | 0.198 ± 0.062 | 198.21  [163.68 – 270.77] | 88.96 [31.75 – 127.43] | 756.79 [472.94 – 1048.78] |
|  | *p* | *0.369* | *0.070* | *0.104* | *0.127* |
| **HT** | No | 0.183 ± 0.064 | 202.46 [136.30 – 271.21] | 63.61 [10.38 – 131.55] | 762.35  [472.29 – 1150.24] |
|  | Yes | 0.198 ± 0.057 | 179.26 [145.10 – 230.58] | 84.10 [52.14 – 118.75] | 613.75 [57.84 – 901.28] |
|  | *p* | *0.363* | *0.247* | *0.680* | *0.145* |
| **DM** | No | 0.186 ± 0.061 | 194.51  [143.29 – 252.64] | 71.89 [25.95 – 127.25] | 762.35 [239.62 – 1039.01] |
|  | Yes | 0.212 ± 0.048 | 187.10  [117.76 – 252.07] | 59.51 [1.01 – 111.45] | 498.90  [0 – 852.09] |
|  | *p* | *0.288* | *0.645* | *0.202* | *0.240* |
| **DL** | No | 0.190 ± 0.060 | 192.66 [138.30 – 235.21] | 68.55 [15.77 – 119.17] | 739.65 [0 – 1004.24] |
|  | Yes | 0.192 ± 0.068 | 195.71 [166.46 – 292.48] | 71.89 [3.85 – 141.09] | 665.63 [238.65 – 971.54] |
|  | *p* | *0.876* | *0.405* | *0.633* | *0.887* |
| **Age** | r | 0.013 | 0.084 | -0.183 | - 0.073 |
|  | *p* | *0.920* | *0.521* | *0.163* | *0.578* |

**ST3.** Association of demographic and clinical features with ApoJ levels. Total ApoJ is expressed as g/L and ApoJ in lipoprotein fractions is expressed as µg of ApoJ per mmol of cholesterol in each lipoprotein. A sample of total plasma with a null value was discarded. M: Male; F: Female: HT: Hypertension; DM: Diabetes Mellitus; DL: Dyslipidemia.

**Supplemental Table 4**

|  | | **Total ApoJ (g/L)** N=59 | **VLDL ApoJ (µg/mmol chol)** N= 60 | **LDL ApoJ (µg/mmol chol)** N=60 | **HDL ApoJ (µg/mmol chol)** N=60 |
| --- | --- | --- | --- | --- | --- |
| **rs11136000** | C | 0.189 ± 0.058 | 189.01  [146.14 – 260.64] | 62.05 [11.04 – 125.20] | 614.68 [0 – 950.47] |
|  | T (MA) | 0.200 ± 0.066 | 200.58  [139.71 – 268.94] | 90.98 [58.70 – 123.61] | 795.87 [586.48 – 1065.01] |
|  | *p* | 0.351 | 0.653 | 0.151 | **0.012 *** |
| **rs7012010** | T | 0.195 ± 0.061 | 190.81 [144.34 – 256.35] | 78.11  [39.50 – 111.87] | 751.23 [330.83 – 1028.20] |
|  | C (MA) | 0.188 ± 0.061 | 194.51 [144.05 – 292.48] | 63.61 [9.72 – 129.29] | 644.44  [0 – 971.54] |
|  | *p* | 0.554 | 0.551 | 0.657 | 0.241 |
| **rs9331888** | C | 0.195 ± 0.063 | 189.01 [142.53 – 267.55] | 72.86 [13.57 – 127.08] | 728.08 [238.65 – 977.89] |
|  | G (MA) | 0.185 ± 0.056 | 202.46 [161.32 – 266.45] | 70.61 [42.22 – 109.06] | 613.75 [13.22 – 995.16] |
|  | *p* | 0.456 | 0.496 | 0.907 | 0.558 |
| **rs9331896** | T | 0.187 ± 0.061 | 189.01 [145.10 – 264.09] | 63.61 [12.31 – 126.14] | 644.44 [13.22 – 961.01] |
|  | C (MA) | 0.203 ± 0.059 | 200.58 [142.53 – 267.55] | 84.10 [46.50 – 122.02] | 795.87 [549.54 – 1065.01] |
|  | *p* | 0.167 | 0.738 | 0.408 | **0.056 ^#^** |

**ST4.** Association of CLU SNPs with ApoJ levels. Total ApoJ is expressed as g/L and ApoJ in lipoprotein fractions is expressed as µg of ApoJ per mmol of cholesterol in each lipoprotein. A sample of total plasma with a null value was discarded. MA: Minor Allele. * p < 0.05; # p < 0.1.

**Supplemental Table 5**

|  | | **Total ApoJ (g/L)** N=40 | **VLDL ApoJ (µg/mmol chol)** N= 40 | **LDL ApoJ (µg/mmol chol)** N=40 | **HDL ApoJ (µg/mmol chol)** N=40 |
| --- | --- | --- | --- | --- | --- |
| **rs11136000** | C | 0.184  [0.149 – 0.242] | 188.70  [144.05 – 233.51] | 62.05  [8.27 – 105.83] | 613.75  [0 – 971.54] |
|  | T (MA) | 0.213  [0.182 – 0.253] | 195.71  [136.30 – 233.80] | 90.98  [58.78 – 111.45] | 849.37  [592.44 – 1065.01] |
|  | *p* | 0.136 | 0.944 | 0.180 | **0.020 *** |
| **rs7012010** | T | 0.205  [0.147 – 0.244] | 187.90  [136.01 – 232.05] | 71.89  [47.87 – 104.94] | 775.89  [35.53 – 1013.02] |
|  | C (MA) | 0.190  [0.155 – 0.244] | 189.82  [149.55 – 232.05] | 65.35  [11.05 – 129.29] | 698.44  [0 – 975.86] |
|  | *p* | 0.867 | 0.450 | 0.983 | 0.719 |
| **rs9331888** | C | 0.205  [0.155 – 0.247] | 187.898  [136.01 – 219.95] | 71.89  [22.54 – 111.45] | 788.84  [25.61 – 1004.09] |
|  | G (MA) | 0.181  [0.147 – 0.236] | 196.63  [151.11 – 258.50] | 66.33  [37.35 – 104.94] | 470.71  [3.30 – 1004.09] |
|  | *p* | 0.248 | 0.462 | 0.992 | 0.434 |
| **rs9331896** | T | 0.181  [0.149 – 0.243] | 183.98  [144.05 – 233.51] | 62.05  [29.54 – 104.94] | 614.68  [0 – 966.28] |
|  | C (MA) | 0.216  [0.190 – 0.248] | 195.71  [136.89 – 225.72] | 87.02  [49.80 – 111.45] | 850.73  [439.07 – 1065.01] |
|  | *p* | 0.111 | 0.872 | 0.343 | **0.045 *** |

**ST5.** Association of CLU SNPs with ApoJ levels in samples obtained >90 days after ICH. Total ApoJ is expressed as g/L and ApoJ in lipoprotein fractions is expressed as µg of ApoJ per mmol of cholesterol in each lipoprotein. MA: Minor Allele. * p < 0.05.

**Supplemental Table 6**

| **CAA** | | **Total ApoE (mg/L)** N=59 | **VLDL ApoE (µmol / mol chol)** N= 60 | **LDL ApoE (µmol / mol chol)**  N= 60 | **HDL ApoE (µmol / mol chol)**  N= 60 |
| --- | --- | --- | --- | --- | --- |
| **Sex** | M | 45.39 ± 19.25 | 345.16 [162.16 – 691.93] | 28.93  [12.20 – 43.99] | 318.35 ± 202.17 |
|  | F | 46.33 ± 15.51 | 274.69  [90.13 – 657.25] | 46.12  [25.74 – 64.51] | 354.98 ± 225.31 |
|  | *p* | *0.839* | *0.524* | *0.056* | *0.532* |
| **HT** | No | 42.75 ± 11.30 | 366.44  [153.65 – 704.21] | 39.99  [19.45 – 68.49] | 353.13 ± 223.92 |
|  | Yes | 50.50 ± 20.51 | 290.92  [159.67 – 634.64] | 35.22  [19.88 – 56.65] | 325.49 ± 215.99 |
|  | *p* | *0.086* | *0.950* | *0.732* | *0.634* |
| **DM** | No | 46.37 ± 16.91 | 393.67  [163.38 – 704.21] | 39.44  [21.69 – 65.46] | 342.40 ± 229.57 |
|  | Yes | 48.03 ± 20.21 | 183.82  [138.69 – 546.96] | 30.32  [14.17 – 58.79] | 347.32 ± 161.28 |
|  | *p* | *0.824* | *0.240* | *0.528* | *0.957* |
| **DL** | No | 46.78 ± 15.36 | 380.06  [154.16 – 696.21] | 39.01  [21.12 – 58.38] | 336.31 ± 241.43 |
|  | Yes | 42.23 ± 20.47 | 273.11  [163.90 – 697.77] | 35.22  [14.99 – 85.01] | 358.35 ± 187.84 |
|  | *p* | *0.363* | *0.677* | *0.965* | *0.731* |
| **Age** | r | -0.028 | -0.084 | -0.243 | 0.048 |
|  | *p* | *0.834* | *0.523* | *0.061* | *0.716* |

**ST6.** Association of demographic and clinical features with ApoE levels. Total ApoE is expressed as mg/L and ApoE in lipoprotein fractions is expressed as µmols of ApoE per mol of cholesterol in each lipoprotein. A sample of total plasma with a null value was discarded. HT: Hypertension; DM: Diabetes Mellitus; DL: Dyslipidemia.

**Supplemental Table 7**

| **CAA** |  | **Total ApoE (mg/L)** N=59 | | **VLDL ApoE**  **(µmol / mol chol)** N= 60 | **LDL ApoE (µmol / mol chol)**  N= 60 | | **HDL ApoE (µmol / mol chol)**  N= 60 | |  |
| --- | --- | --- | --- | --- | --- | --- | --- | --- | --- |
| **APOEε2** | No | | 43.16 ± 14.02 | 287.58 [143.76 – 634.64] | | 35.07 [17.64 – 51.61] | | 323.57 ± 211.25 | |
|  | Yes | | 67.04 ± 21.56 | 407.84 [200.08 – 908.61] | | 67.48 [58.79 – 171.32] | | 477.68 ± 219.04 | |
|  | *p* | | **<*0.001 ****** | *0.377* | | ***0.001 ***** | | *0.076* **^#^** | |
| **APOEε4** | No | | 48.59 ± 17.17 | 363.52 [151.26 – 697.77] | | 38.57 [20.21 – 67.48] | | 355.04 ± 228.17 | |
|  | Yes | | 36.82 ± 11.73 | 284.09 [120.15 – 504.03] | | 36.76 [20.33 – 46.34] | | 292.80 ± 163.54 | |
|  | *p* | | ***0.024 **** | *0.641* | | *0.484* | | *0.363* | |

**ST7.** Association of ApoE genotype with ApoE levels. Total ApoE is expressed as mg/L and ApoE in lipoprotein fractions is expressed as µmols of ApoE per mol of cholesterol in each lipoprotein. A sample of total plasma with a null value was discarded. * p < 0.05; ** p < 0.01; *** p < 0.001; # p < 0.1.

**Supplemental Table 8**

| **CAA** |  | **Total ApoE (mg/L)** N=40 | | **VLDL ApoE**  **(µmol / mol chol)** N= 40 | **LDL ApoE (µmol / mol chol)**  N= 40 | | **HDL ApoE (µmol / mol chol)**  N= 40 | |  |
| --- | --- | --- | --- | --- | --- | --- | --- | --- | --- |
| **APOEε2** | No | | 44.80  [33.50 – 53.80] | 246.47  [149.22 – 616.76] | | 32.43  [20.04 – 64.51] | | 333.69 ± 210.54 | |
|  | Yes | | 61.50  [50.83 – 81.40] | 580.46  [233.83 – 911.02] | | 93.13  [56.72 –176.51 ] | | 491.43 ± 236.61 | |
|  | *p* | | **0.012*** | 0.271 | | **0.005**** | | 0.104 | |
| **APOEε4** | No | | 47.10  [38.28 – 59.70] | 327.22  [149.22 – 719.94] | | 39.28  [22.93 – 91.08] | | 382.93 ± 225.26 | |
|  | Yes | | 34.30  [29.73 – 37.63] | 266.97  [151.45 – 432.72] | | 35.41  [19.25 – 46.00] | | 212.39 ± 96.62 | |
|  | *p* | | **0.007**** | 0.493 | | 0.447 | | **0.007**** | |

**ST8.** Association of ApoE genotype with ApoE levels in chronically obtained samples (>90 days after ICH). Total ApoE is expressed as mg/L and ApoE in lipoprotein fractions is expressed as µmols of ApoE per mol of cholesterol in each lipoprotein. * p < 0.05; ** p < 0.01.
